# Design, synthesis, *in vitro* inhibition and toxicological evaluation of human carbonic anhydrases I, II and IX inhibitors in 5-nitroimidazole series

**DOI:** 10.1080/14756366.2019.1685510

**Published:** 2019-11-05

**Authors:** Ashok Aspatwar, Nanda Kumar Parvathaneni, Harlan Barker, Emilie Anduran, Claudiu T. Supuran, Ludwig Dubois, Philippe Lambin, Seppo Parkkila, Jean-Yves Winum

**Affiliations:** a Faculty of Medicine and Health Technology, Tampere University, Tampere, Finland; b Department of Precision Medicine, The M-Lab, GROW – School for Oncology and Developmental Biology, Maastricht Comprehensive Cancer Centre, Maastricht University Medical Centre, Maastricht, The Netherlands; c Institut des Biomolécules, Max Mousseron (IBMM) UMR 5247 CNRS, ENSCM, Université de Montpellier, Bâtiment de Recherche Max Mousseron, Ecole Nationale Supérieure de Montpellier, Montpellier, France; d NEUROFARBA Department, Section of Pharmaceutical and Nutraceutical Sciences, University of Florence, Polo Scientifico, Firenze, Italy; e Fimlab Ltd and Tays Cancer Center, Tampere University Hospital, Tampere, Finland

**Keywords:** Carbonic anhydrases, carbonic anhydrase inhibitors, synthesis, toxicity evaluation, lethal concentration, zebrafish embryos

## Abstract

With the aim to obtain novel compounds possessing both strong affinity against human carbonic anhydrases and low toxicity, we synthesised novel thiourea and sulphonamide derivatives **3**, **4** and **10**, and studied their *in vitro* inhibitory properties against human CA I, CA II and CA IX. We also evaluated the toxicity of these compounds using zebrafish larvae. Among the three compounds, derivative **4** showed efficient inhibition against hCA II (KI = 58.6 nM). Compound **10** showed moderate inhibition against hCA II (KI = 199.2 nM) and hCA IX (KI = 147.3 nM), whereas it inhibited hCA I less weakly at micromolar concentrations (KI = 6428.4 nM). All other inhibition constants for these compounds were in the submicromolar range. The toxicity evaluation studies showed no adverse effects on the zebrafish larvae. Our study suggests that these compounds are suitable for further preclinical characterisation as potential inhibitors of hCA I, II and IX.

## Introduction

Carbonic anhydrases (CAs, EC 4.2.1.1) are metalloenzymes, present in most of the living organisms and encoded by seven evolutionarily unrelated gene families: the α-.β-.γ-.δ-.ζ-.η-. and θ-CAs^1^. The CAs catalyse the basic reaction of reversible hydration of carbon dioxide to bicarbonate and proton^2^. This reaction is fundamental for biochemical processes in all living organisms^1^^,^^3^. Invertebrates and lower organisms contain all the seven members of CA gene families and, in some cases, multiple gene families with in the same organism^1^. In contrast, vertebrates and mammals contain only α-CAs^1^. In humans, the CAs regulate a broad range of physiological processes, such as respiration, transport of carbon dioxide/bicarbonate between the lung and metabolising tissues, pH homeostasis, and electrolyte secretion in many tissues and organs^2^. There are 15 CA isoforms in humans of which 12 are catalytically active. Three are inactive due to the lack of one or more of the three-histidine residues required for enzymatic activity^2–3^. The human CA isoforms differ from one another in cellular localisation, distribution in organs, levels of expression in tissues and their kinetic properties^4–5^. The human CA isoforms have high sequence and structural similarities, and therefore only subtle differences exist in their active sites^6–7^.

In humans, increased expression of CA isoforms is sometimes associated with certain disease conditions^2^. The cytosolic hCA I and II proteins have been linked to retinal and cerebral oedema, glaucoma, epilepsy and altitude sickness^2^^,^^4^. hCA IX, a transmembrane CA isoform, is overexpressed under hypoxic conditions in solid tumours and is involved in the progression of cancer^8^^,^^9^. Many CA inhibitors have been used for treating various human diseases^2^. Even if the inhibitors achieve desirable therapeutic effects, they may cause unacceptable level of toxicity in humans^10^^,^^11^, most probably due to the off-target effects attributable to the inhibition of CA isoforms that are not involved in the target disease or due to general toxic effects of the chemical^12^^,^^13^. Therefore, to target particular therapeutic areas with minimal off-target effects, inhibition of specific enzymes is necessary. Thus, there is a particular need to develop inhibitors that are not only selective against the human CAs but are also safe for clinical use^14^.

Along this line, we have previously developed numerous potent CA inhibitors that are not only effective against specific human α-CAs and mycobacterial β-CAs, but also show minimal toxicity and are suitable for developing further for clinical use as drugs^14–16^. Recently, we reported sulphonamide and carbamate compounds as hCA IX and mycobacterial β-CA inhibitors^14–16^. In addition to the synthesis and *in vitro* and *in vivo* evaluation of these compounds, we also tested them for safety and toxicity using 1–5 d post-fertilisation (dpf) zebrafish larvae. Our studies showed that these inhibitors are safe for further preclinical characterisation using *in vivo* models^14–16^.

In addition, in this study, we were interested in incorporating a nitro-imidazole moiety as a privileged hypoxia sensitising scaffold in the structure of CA inhibitors. The rationale was to investigate a strategy of dual targeting (hypoxia and hCA IXn/hCA XII) in the context of anticancer agents^17^. Previous results have shown the validity of this approach when conjugation of 5-nitroimidazole derivatives to CA inhibitors structures led to new radiosensitiser agents targeting hypoxic tumours^17–20^. To further investigate the effect and the influence of the 5-nitro imidazole moiety and develop new potent and safer inhibitors, we have been continuing our research on new inhibitors in the nitroimidazole series. In the present study, we report the design, synthesis, *in vitro* CA inhibition, and evaluation of toxicity of novel thiourea derivatives **3** and **4** and a sulphonamide derivative **10** using zebrafish larvae.

## Materials and methods

### Chemistry

All reagents and solvents were of commercial quality and used without further purification unless otherwise specified. All reactions were carried out under an inert atmosphere of nitrogen. TLC analyses were performed on silica gel 60 F254 plates (Merck Art. no. 1.05554). Spots were visualised under 254 nm UV illumination or by ninhydrin solution spraying. Melting points were determined on a Büchi Melting Point 510 and are uncorrected. ^1^H and ^13^C NMR spectra were recorded on Bruker DRX-400 spectrometer using DMSO-d_6_ as solvent and tetramethylsilane as internal standard. For ^1^H NMR spectra, chemical shifts are expressed in δ (ppm) downfield from tetramethylsilane and coupling constants (*J*) are expressed in Hertz. Electron ionisation mass spectra were recorded in positive or negative mode on a Water MicroMass ZQ. All compounds that were tested against purified physiological isoforms of CAs were analysed by high-resolution ESI mass spectra (HRMS) using on a Q-ToFI mass spectrometer fitted with an electrospray ion source to confirm the purity of >95%.

*1-(2-Isothiocyanatoethyl)-2-methyl-5-nitro-1H-imidazole (****2****):* Thiophosgene (1 equiv.) and sodium hydroxide (2 equiv.) were added to a solution of **1** (0.5 equiv.) in chloroform and water (4:1) at 0 °C, and reaction mixture was warmed to room temperature. Thin layer Chromatography confirmed complete disappearance of the starting material. Reaction mixture was then washed with water and extracted with DCM. The organic layer was dried over anhydrous sodium sulphate, filtered and evaporated to get crude compound which was purified by chromatography on silica gel using ethyl acetate and petroleum ether as eluent with a gradient from 5:5 to 9:1. Yield: 64%, ^1^H NMR (400 MHz, DMSO-d_6_) *δ* 8.10 (s, 1H), 4.65–4.60 (m, 2H), 4.17–4.12 (m, 2H), 3.35 (s, 3H). ^13^C NMR (101 MHz, DMSO-d_6_) *δ* 151.80, 138.80, 133.65, 129.62, 45.06, 14.28. MS (ESI^+^) *m/z* 213.04 [M + H]^+^.

*4-(2-(3-(2-(2-Methyl-5-nitro-1H-imidazol-1-yl)ethyl)thioureido)ethyl)phenyl sulphamide (****3****):* 4-(2-aminoethyl) aniline (1 equiv.) was added to a solution of **2** (1 equiv.) in acetonitrile and allowed to stir at room temperature overnight. Reaction mixture was washed with water and extracted with ethyl acetate. The organic layer was dried over anhydrous sodium sulphate, filtered and evaporated under vacuum to get crude compound which was then reacted as-is with sulphamoyl chloride (3 equiv.) in dimethylacetamide (DMA). After stirring for one night at room temperature, the mixture was diluted with ethyl acetate, and washed three times with water. The organic layer was dried over anhydrous magnesium sulphate and concentrated under vacuum. The residue was purified by chromatography on silica gel using methylene chloride-methanol 9:1 as eluent. Yield: 43%, ^1^H NMR (400 MHz, DMSO-d_6_) *δ* 9.35 (s, 1H), 8.03 (s, 1H), 7.51 (s, 2H), 7.11 (d, *J* = 9.1, 4H), 7.01 (s, 2H), 4.41 (s, 2H), 3.81 (s, 2H), 2.68 (s, 2H), 2.36 (s, 3H). ^13^C NMR (101 MHz, DMSO-d_6_) *δ* 151.98, 139.08, 138.13, 133.70, 133.45–133.11, 129.40, 118.95, 55.38, 45.93, 14.30. MS (ESI^+^) *m/z* 428.12 [M + H]^+^.

*4-(2-(3-(2–(2-methyl-5-nitro-1H-imidazol-1-yl)ethyl) thioureido)ethyl)phenyl sulphamate* ***(4):*** The same protocol as for the synthesis of compound **3** starting from **2**. Yield: 60%, ^1^H NMR (400 MHz, DMSO-d_6_) *δ* 8.03 (s, 1H), 7.95 (s, 2H), 7.58 (d, *J* = 38.2, 2H), 7.29 (d, *J* = 8.5, 2H), 7.20 (d, *J* = 8.5, 2H), 4.42 (s, 2H), 3.82 (s, 2H), 2.77 (s, 2H), 2.36 (s, 3H). ^13^C NMR (101 MHz, DMSO-d_6_) *δ* 151.44, 148.56, 138.56, 137.61, 133.16, 129.77, 121.97, 45.39, 30.63, 13.75. MS (ESI^+^) *m/z* 429.10 [M + H]^+^.

*2-(2-Methyl-5-nitro-1H-imidazol-1-yl) ethyl methanesulphonate (****6****):* Methane sulphonylchloride (1.1 equiv.) was added dropwise to a stirred solution of metronidazole, **5** (1 equiv.), triethylamine (2 equiv.) and DMAP (1.1 equiv.) in anhydrous methylene chloride at 0 °C. The reaction mixture was allowed to warm to room temperature and stirred for overnight. The reaction mixture was diluted with methylene chloride and washed with water and brine. The organic layer was dried over anhydrous sodium sulphate, filtered and evaporated to get crude compound which was used further without purification. Yield: quantitative. ^1^H NMR (DMSO-d_6_, 400 MHz) *δ* 8.05 (s, 1H), 4.61 (t, *J* = 7.3, 2H), 3.51 (t, *J* = 7.3, 2H), 2.50 (s, 3H); ^13^C NMR (DMSO-d_6_, 101 MHz) δ 151.08, 138.18, 133.14, 47.09 (s, 1H), 14.09, 2.28. MS (ESI^+^) *m/z* 281.97 [M + H]^+^.

*S-(2-(2-Methyl-5-nitro-1H-imidazol-1-yl)ethyl) ethanethioate (****7****):* Potassium thioacetate (2 equiv.) was added to a solution of **6** (1 equiv.) in anhydrous DMF and stirred at room temperature for 16 h. The reaction mixture was then diluted with ethyl acetate and washed with water and brine. The organic layer was dried over anhydrous sodium sulphate, filtered and concentrated under vacuum to give thioacetate **7**. Yield: 83%, ^1^H NMR (400 MHz, DMSO-d_6_) δ 8.01 (s, 1H), 4.45 (t, *J* = 6.6, 2H), 3.27 (t, *J* = 6.6, 2H), 2.46 (s, 3H), 2.29 (s, 3H). ^13^C NMR (101 MHz, DMSO) δ 194.59, 151.25, 138.42, 132.92, 44.68, 30.32, 27.87. MS (ESI^+^) *m/z* 230.06 [M + H]^+^.

*2-(2-Methyl-5-nitro-1H-imidazol-1-yl)ethane-1-sulphonyl chloride (****9****):* A solution of **7** (1 equiv.) was treated with hydrogen peroxide [35% w/w in water (6 ml)] and acetic acid (7 ml). The reaction mixture was allowed to stir for 24 h at room temperature; the excess hydrogen peroxide was quenched by addition of 10% Pd/C (0.050 g). The mixture was filtered on celite, concentrated, and co-evaporated with toluene. The obtained sulphonic acid was used further without purification. The crude 2-(2-methyl-5-nitro-1*H*-imidazol-1-yl) ethane-1-sulphonic acid **8** (1 equiv.) was suspended in anhydrous DCM and a solution of phosgene in toluene (20% wt., 3.5 ml) and dry DMF (1 ml) was added under argon atmosphere. After 1 h an additional 1 ml phosgene solution was added, and the mixture was stirred for 2 h under same conditions. After precipitation, the reaction mixture was filtered to get pure compound **9** as solid. Yield: 66%, ^1^H NMR (400 MHz, DMSO-d_6_) *δ* 8.80 (s, 1H), 4.63 (t, *J* = 6.3, 2H), 2.92 (t, *J* = 6.3, 2H), 2.78 (s, 3H), ^13^C NMR (101 MHz, DMSO-d_6_) *δ* 149.90, 138.05, 123.91, 48.67, 44.17, 12.13. MS (ESI^+^) *m/z* 236.00 [M + H]^+^.

*2-(2-Methyl-5-nitro-1H-imidazol-1-yl)ethane-1-sulphonamide (****10****):* To a solution of compound **9** (1 equiv.) in toluene was added at room temperature 0.5 M solution of liq. ammonia in 1,4-dioxane (2 ml). After 1 h, the reaction mixture was filtered to afford pure compound as white solid. Yield: 68%, ^1^H NMR (400 MHz, DMSO-d_6_) *δ* 8.03 (s, 1H), 7.39 (s, 2H), 4.63 (t, *J* = 7.0, 2H), 3.49 (t, *J* = 7.0, 2H), 2.49 (s, 3H). ^13^C NMR (101 MHz, DMSO-d_6_) *δ* 151.43, 138.36, 133.04, 53.08, 40.85, 13.94. MS (ESI^+^) *m/z* 235.05 [M + H]^+^.

### *In vitro* inhibition studies

An Applied Photophysics stopped-flow instrument was used for assaying the CA-catalyzed CO_2_ hydration activity^21^. Phenol red (at a concentration of 0.2 mM) was used as an indicator, working at the absorbance maximum of 557 nm, with 20 mM Hepes (pH 7.5) as buffer and 20 mM Na_2_SO_4_ (for maintaining the ionic strength constant), following the initial rates of the CA-catalysed CO_2_ hydration reaction for a period of 10–100 s. The CO_2_ concentrations ranged from 1.7 to 17 mM for the determination of the kinetic parameters and inhibition constants. For each inhibitor, at least six traces of the initial 5–10% of the reaction were used for determining the initial velocity. The uncatalysed rates were determined in the same manner and subtracted from the total observed rates. Stock solutions of inhibitor (0.1 mM) were prepared in distilled-deionized water, and dilutions up to 0.01 nM were done thereafter with distilled-deionized water. Inhibitor and enzyme solutions were pre-incubated together for 15 min at room temperature prior to assay to allow for the formation of the E-I complex. The inhibition constants were obtained by non-linear least-squares methods using PRISM 3 and represent the average from at least three different determinations. CA isoforms were recombinant ones obtained in house as reported earlier.

### Toxicity evaluation

#### Inhibitors

The three compounds **3**, **4** and **10** were designed and synthesised with the purpose of developing them as inhibitors of human CAs. Subsequently, all the three compounds were investigated *in vitro* as inhibitors of human CA I, CA II and CA IX. These compounds were dissolved in dimethyl sulphoxide (DMSO) (Sigma-Aldrich, St. Louis, MO) to prepare 100 mM stock solutions. Before start of each experiment, the series of dilatation were made from the above stock in an embryonic medium [5.0 mM NaCl, 0.17 mM KCl, 0.33 mM CaCl_2_, 0.33 mM MgSO_4_, and 0.1% w/v Methylene Blue (Sigma-Aldrich, Darmstadt, Germany)]. Embryos after 1-day post fertilisation were exposed the each of the diluted inhibitor solution.

### Maintenance of zebrafish

AB strains of wild-type adult zebrafish were maintained at 28.5 °C in an incubator^15^. For collecting embryos, 3–5 pairs of male and female fish were set up overnight for breeding^22^. The next morning, from the overnight breeding tanks 1–2-h post fertilisation (hpf) embryos were collected in a sieve and rinsed with embryonic medium [5.0 mM NaCl, 0.17 mM KCl, 0.33 mM CaCl_2_, 0.33 mM MgSO_4_, and 0.1% w/v Methylene Blue (Sigma-Aldrich, Darmstadt, Germany)] and kept the collected embryos in an incubator at 28.5 °C overnight. The toxicity evaluation studies of the inhibitors were performed using the fish that were 24 hpf. All the zebrafish experiments were performed at the zebrafish core facility, Tampere University, Finland and according to the protocol used in our laboratory^22^.

### Ethical statement

Tampere University has an established zebrafish core facility authorisation granted by the National Animal Experiment Board (ESAVI/7975/04.10.05/2016). The experiments using developing zebrafish embryos were performed according to the Provincial Government of Eastern Finland Province Social and Health Department Tampere Regional Service Unit protocol # LSLH-2007–7254/Ym-23. Care was taken to ameliorate suffering by euthanizing the 5 dpf larvae by prolonged immersion in a petri dish containing an overdose of Tricaine (Sigma-Aldrich, St. Louis, MO) before fixing in buffered formaldehyde for histochemical analysis.

### Determination of LC_50_

The LC_50_ values of the compounds **3**, **4** and **10** were determined using 1-dpf embryos exposed to 10–12 different inhibitor concentrations. For each concentration of the inhibitor, we used 30 1dpf larvae^13^^,^^22^. In each group, the larvae were exposed to different concentrations of the inhibitors that ranged from 12.5 µM to 3 mM. The dose response curve (DRC) was calculated using DRM of the DRC R package^23^. The control group was constituted of an equal number of larvae not treated with any chemical and the larvae that were treated with 1% of DMSO. Toxicological evaluation studies were performed in 24-well plates (Corning V R Co-star V R cell culture plates). In each well, we placed 1–2 1-dpf embryos in 1 ml of embryonic medium containing a diluted inhibitor. For control groups, 1% DMSO diluted in either embryonic medium or embryonic medium with no chemical compound. A minimum of three sets of experiments were carried out for each inhibitor. The mortality of the larvae was checked every 24 h until 5 d after exposure to the inhibitors.

### Phenotypic analysis of control and inhibitor treated larvae

We assessed the effect of inhibitors on the zebrafish larvae after 5 d of exposure to the drugs; we analysed eight observable phenotypic parameters: (1) mortality, (2) hatching, (3) oedema, (4) movement pattern, (5) yolk sack utilisation, (6) heartbeat, (7) body shape and (8) swim bladder development, using a stereo microscope and recorded the observations for each inhibitor treated and control groups. The images of the developing larvae were taken using a Lumar V1.12 fluorescence microscope attached to a camera with a 1.5 lens (Carl Zeiss MicroImaging GmbH, Göttingen, Germany). The images were analysed with AxioVision software versions 4.7 and 4.8 as described in our standard protocol for assessment of toxicity and safety of the chemical compounds^22^.

### Swim pattern analysis

The movement of the zebrafish larvae was tracked from day 4 of exposure to these inhibitors. Similarly, detailed analysis of the swim pattern of the larvae was performed at the end of day 5 after exposure to the inhibitors. For analyses of movement pattern, about 10–15 the zebrafish larvae were placed in a 35 mm × 15 mm petri dish containing embryonic medium and the larvae were allowed to settle in the petri dish for 1 min. The movement of the zebrafish larvae was observed under the microscope for 1 min. A short video of about 1 min was taken for each group of larvae that were treated with 125 µM concentration. The swim patterns were compared with the control group zebrafish larvae that were not treated with any inhibitor.

### Histological studies

The histological analyses were done to check the effect of inhibitors on the internal tissues of the larvae that were treated with different concentrations of the inhibitors used in the study and the control group larvae not treated with any inhibitor and the larvae treated with 1% DMSO. After 5 d of exposure to different concentration of the inhibitors, the larvae were washed with phosphate buffered saline (PBS) immersed in excess amounts of Tricaine to anaesthetize the larvae. The larvae thus prepared were transferred to a 1.5 ml microcentrifuge tube and fixed in buffered formaldehyde (formaldehyde solution 4%, pH 6.9) in PBS for 3 h at room temperature or overnight at 4 °C. After the fixation, the larvae were transferred to 70% ethanol and stored at 4 °C before being embedded in paraffin. The paraffin-embedded samples were sectioned into 5 µM thin slices for the histochemical staining. The fixed sections containing samples were deparaffinised in xylene, rehydrated in an alcohol series, and stained with Mayer's Haematoxylin and Eosin Y (both from Sigma-Aldrich, Darmstadt, Germany). After dehydration, the slides were mounted with Entellan^®^ Neu (Merck; Darmstadt, Germany). The slides containing the tissues were examined for morphological changes in the tissues of the larvae exposed to the drugs and photographed using a Nikon Microphot microscope (Nikon Microphot- FXA, Tokyo, Japan). All the procedures were carried out at room temperature unless stated otherwise.

### Statistical analysis

The GraphPad Prism software (5.02) (GraphPad Software, La Jolla, CA) was used to perform some of the statistical analyses. For statistical analysis of the toxicity parameters, a two-tailed Fisher’s test was used and *p* values below 0.05 were considered significant.

## Results and discussion

### Chemistry

**Scheme 1.**
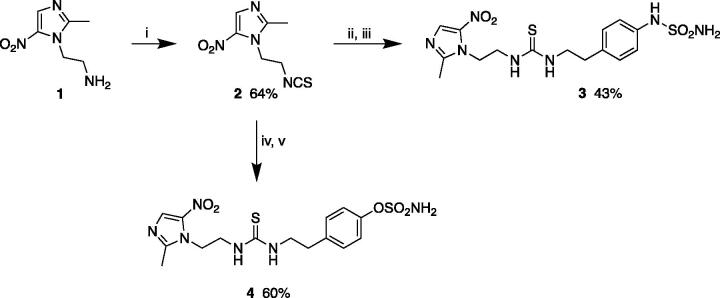
Synthesis of thioureas **3** and **4**. Reagents and conditions: (i) thiophosgene, NaOH, CHCl_3_, 0 °C-rt; (ii) 4-(2-aminoethyl) aniline, CH_3_CN, rt; (iii) NH_2_SO_2_Cl, DMA, rt; (iv) 4-(2-aminoethyl) phenol, CH_3_CN, rt; (v) NH_2_SO_2_Cl, DMA, rt.

Thiourea derivatives **3** and **4** were prepared starting from the amino analogue of metronidazole **1** which was converted to isothiocyanate **2** using thiophosgene under basic conditions. Reaction either with 4-(2-aminoethyl) aniline or 4-(2-aminoethyl) phenol in acetonitrile led to the thiourea-derivatives which were then sulphamoylated with sulphamoyl chloride to give, respectively, sulphamide **3** and sulphamate **4** (Scheme 1)^24^^,^^25^.

**Scheme 2.**
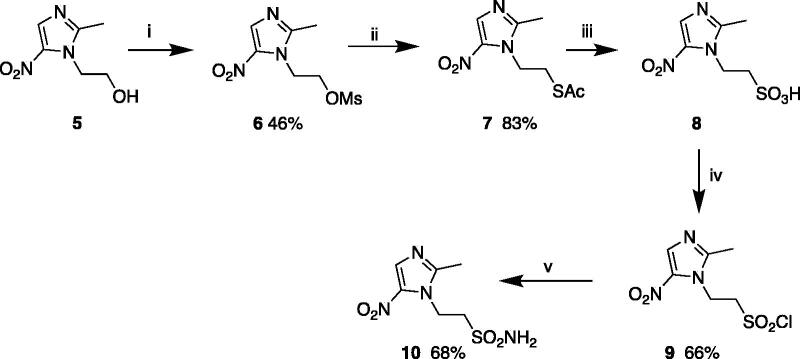
Synthesis of sulphonamide **10**. Reagents and conditions: (i) CH_3_SO_2_Cl, NEt_3_, DMAP, DCM, 0 °C – rt; (ii) KSAc, DMF, rt; (iii) 35% H_2_O_2_-CH_3_COOH, rt; (iv) phosgene, cat. dry DMF, dry DCM, rt, (v) NH_3_ in 1,4 dioxane, benzene, rt.

Compound **10** was prepared in a five-step synthesis starting from metronidazole **5.** Mesylation of **5** using methane sulphonyl chloride under basic conditions led to compound **6.** Reactions with potassium thioacetate followed by oxidation and treatment with phosgene allowed the formation of sulphonyl chloride **9**. Finally, compound **10** was obtained by treatment with a solution ammonia in 1,4-dioxane at room temperature (Scheme 2)^26^.

### CA inhibition assay

All compounds reported here were assayed as inhibitors of three physiologically relevant CA isoforms, the cytosolic hCA I and II and the transmembrane, tumour-associated hCA IX using the CO_2_ hydrase assay (Table 1)^17^. The clinically employed sulphonamide, acetazolamide (**AAZ**, 5-acetamido-1,3,4- thiadiazole-2-sulphonamide), has been used as a standard in these measurements.

**Table 1.** Inhibition data of compounds **3**, **4** and **10** against hCA I, hCA II and hCA IX using a stopped flow CO_2_ hydrase assay.

Compound	*K_I_* (nM)^a^			Selectivity ratios
	hCA I	hCA II	hCA IX	*K_I_*hCA II/*K_I_*hCA IX
**3**	152.2	612.8	290.6	2.10
**4**	127.8	58.6	642.6	0.09
**10**	6428.4	199.2	147.3	1.35
**AAZ**	250	12.0	25	0.48

^a^ Average from three different assays, by a stopped flow technique (errors were in the range of ± 5**–**10% of the reported values).

Compound **10** showed very weak inhibition potency against the abundant cytosolic hCA I with a *K_I_*value of 6428 nM. The thiourea derivatives **3** and **4** demonstrated sub-micromolar inhibition potency against all CA isoforms. Compound **4** showed the most efficient binding to hCA II (*K_I_*= 58.6 nM) with a selectivity ratio of 0.09 for inhibiting hCA II over hCA IX, explained by the influence of the substitution on the benzene ring. Compound **10**, the sulphonamide derivative of the sulphamide and sulphamate analogues previously reported and biologically evaluated by our group^24^, showed moderate binding efficacy towards hCA II (*K_I_*= 199.2 nM) and hCA IX (*K_I_*= 147.3 nM). The better binding efficacy of sulphonamide **10** compared to the sulphamide and sulphamate compounds might be explained by higher stability under acidic conditions, i.e. sulphonamide > sulphamide > sulphamate.

## Evaluation of safety and toxicity of compounds 3, 4 and 10

### Determination of inhibitor half maximal lethal concentration (LC_50_)

**Figure 1.**
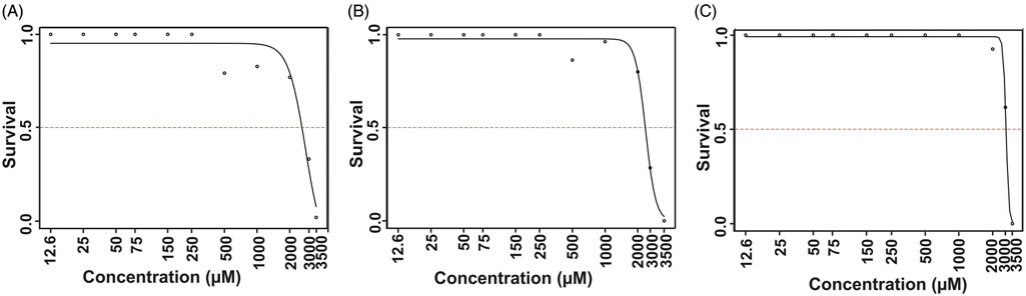
The LC_50_ values of the tested CA inhibitor compounds. The LC_50_ doses for the inhibitors were determined based on the 50% mortality of the larvae at the end of 5 d after the exposure of embryos to different concentrations of inhibitors: compound **3** (A), **4** (B) and **10** (C). The LC_50_ doses were determined after three independent experiments with similar experimental conditions (for each compound *N* = 90).

The lethal effects of all three inhibitor compounds were concentration dependent on the developing zebrafish embryos that were exposed to the inhibitor for 5 d (Figure 1). None of the three inhibitors exhibited any significant mortality in up to 1.5 mM concentration at the end of 5 d of exposure to the inhibitors. Significant mortality of zebrafish larvae emerged at higher concentrations (>2 mM), and the LC_50_ values at the end of five days were 3 mM for **3** and 2.6 mM for **4** and **10**. The half maximal lethal concentrations of the inhibitors were higher compared to any toxicity and safety assessment studies that we have performed earlier^14–16^, suggesting that these compounds can be further developed as inhibitors of human CA I, CA II and CA IX.

### Phenotypic analyses of zebrafish larvae treated with inhibitor compounds

**Figure 2.**
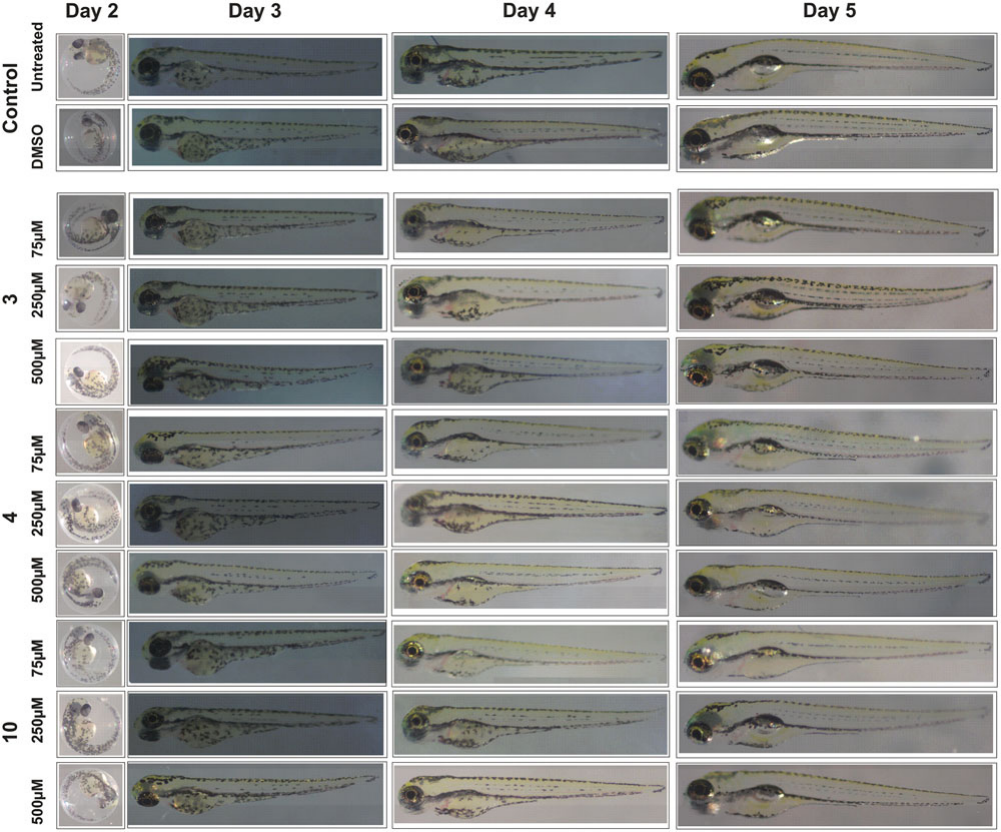
The images of zebrafish larvae in control and inhibitor treated groups. Representative images of 2–5 dpf zebrafish larvae exposed to different concentrations (75, 250 and 500 μM) of compound **3**, **4** and **10**. The images of control group (not treated with inhibitors) and 1% DMSO treated zebrafish larvae show normal development (*n* = 60).

To assess toxic effects of the inhibitors on the developing zebrafish larvae we analysed eight observable phenotypic parameters namely: hatching, heartbeat, yolk sack utilisation, movement pattern, body shape, swim bladder development, mortality and oedema. The parameters were monitored using a stereo microscope and observations were recorded for each group in comparison with control embryos that were not treated with any of the compounds or treated with 1% DMSO. The images in Figure 2 show the representative larvae exposed to the compounds with concentrations ranging from 75 µM to 500 µM as compared to the control group larvae. The microscopic examination did not show any of the observable changes in the developing zebrafish embryos at these concentrations and the embryos showed normal phenotypes throughout the period of embryonic development from 2 to 5 dpf.

**Figure 3.**
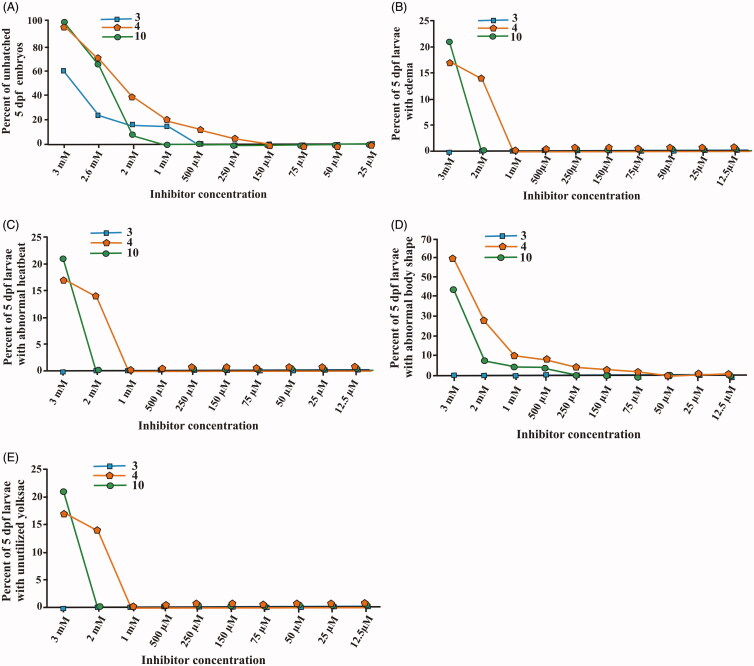
The effect of compounds **3**, **4** and **10** CA inhibitors on the phenotypic parameters of the 5 dpf zebrafish larvae. The plot graphs of different phenotypic abnormalities in (A) hatching, (B) oedema, (C) heartbeat (D) body shape and (E) yolk sac utilisation. For each concentration, *n* = 90. *p < 0.05 by two-tailed Fisher’s test.

We further assessed the toxicity of compounds **3**, **4** and **10** compounds in zebrafish larvae across the concentration range 12 µM–3 mM. Figure 3 presents plots of dose-dependent effects of inhibitors on different parameters in treated larvae. The results indicated that none of the inhibitors show significant toxic effects at submillimolar range. Previous toxicity studies on hCA IX and mycobacterial β-CA inhibitors showed significant effects on larval mortality and observable phenotypic parameters in the concentration range of 50–300 µM^14^^,^^16^. Therefore, our present results show that **3**, **4** and **10** are less toxic in the zebrafish larval model.

### Histochemical analysis

**Figure 4.**
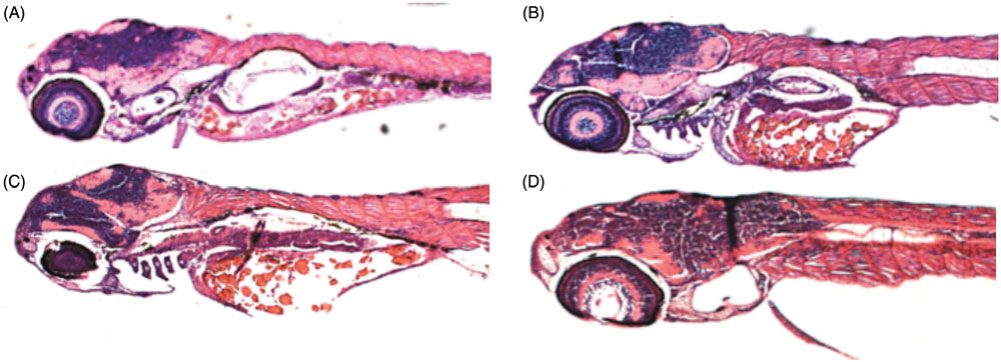
Histochemical analyses of zebrafish larvae. Representative sagittal sections of an untreated zebrafish larva (A) or larva treated with 500 μM of compound **3** (B), 4 (C) or **10** (D). The images presented here are selected from three independent groups of experiments.

The effects of different concentrations of CA inhibitors on histology were studied by analysing the sections of 5 dpf zebrafish larvae stained with haematoxylin and eosin (H & E). The stained sagittal sections of both the inhibitor treated and control larvae were compared by light microscopy. Representative images of zebrafish larval sections are shown in Figure 4. The histological examination revealed no damage to the tissues of the developing zebrafish larvae. Even high concentrations (up to 2 mM) showed no apparent damage to the internal tissues, suggesting that these compounds are safe and can be further developed as CA-specific inhibitors for clinical use.

### Analysis of swim pattern

During development, zebrafish embryos are easily affected by chemical compounds compared to adult zebrafish or other animal or cell models, and therefore are suitable for assessing the subtle toxic effects of the chemicals^14^^,^^22^. To assess the subtle toxic effects of the inhibitors, the swim pattern of the larvae was examined under the microscope during treatment with the CA inhibitors. The swim pattern analysis showed no abnormal or ataxic movement pattern in the larvae exposed to the inhibitors, up to 2 mM concentration. However, a detailed analysis of the videos at the end of 5 d of inhibitor exposure showed that the larvae of the inhibitor treated groups (125 µM) were not as active and tended to remain at the sides of petri dish compared to the control group larvae (Representative video of swim pattern treated with compound **3**: Supplementary Appendix A). The results of the current study reveal that the larvae treated with **3**, **4** and **10** become slower while swimming as compared to the swim pattern of the control group larvae. However, swim pattern of larvae treated with **3**, **4** and **10** was unlike the larvae treated with nitroimidazole-based inhibitors DTP338 and DTP348, which showed ataxic movement pattern due to neurotoxicity at a concentration as low as 100 µM^14^. Therefore, these novel thiourea and sulphonamide derivatives can be considered safer for further preclinical characterisation and development as potential drugs.

## Conclusions

In conclusion, we have designed and synthesised thiourea and sulphonamide derivative compounds with the potential to inhibit human CA I, CA II and CA IX. These proteins which play important pathophysiological roles in human diseases, such as well-known role of CA IX in the progression of solid tumours, and those of CA I and CA II in retinal and cerebral oedema, glaucoma, epilepsy and altitude sickness. The inhibition kinetics of these inhibitors were studied against recombinant human hCA I, hCA II and hCA IX *in vitro*. Among these compounds, **4** was the most efficient against hCA II (*K_I_* 58.6 nM). **10** inhibited human CA IX with *K_I_* 147.3 nM. The inhibition properties of these inhibitors against the three hCA isoforms prompted us to test these drugs further for their safety in a zebrafish larval model.

The toxicological evaluation indicated that these inhibitors showed no adverse morphological changes nor did they cause abnormal swim pattern at any sub-millimolar concentration. Taken together, the results from both *in vitro* inhibition studies and toxicological evaluation, we suggest that these inhibitors could be developed into potential drugs targeting hCA I, hCA II and hCA IX with no, or minimal, off-target effects.

## Supplementary Material

Supplemental Material
