# Supplementary material for: Design, synthesis, in vitro inhibition and toxicological evaluation of human carbonic anhydrases I, II and IX inhibitors in 5-nitroimidazole series
Source: J Enzyme Inhib Med Chem. 2019 Nov 5;35(1):109–17. doi: 10.1080/14756366.2019.1685510 (PMC6844379; doi:10.1080/14756366.2019.1685510)

Supplementary data

^{1}H NMR spectra of compound **4**

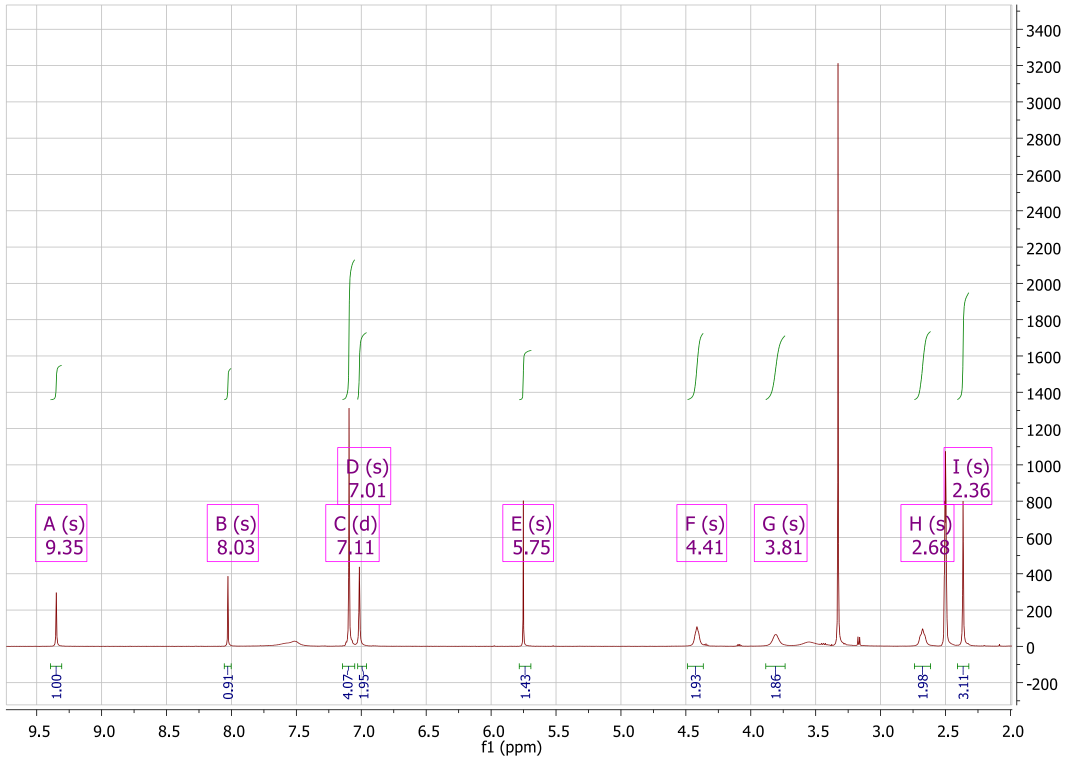

^{13}C NMR spectra of compound **4**

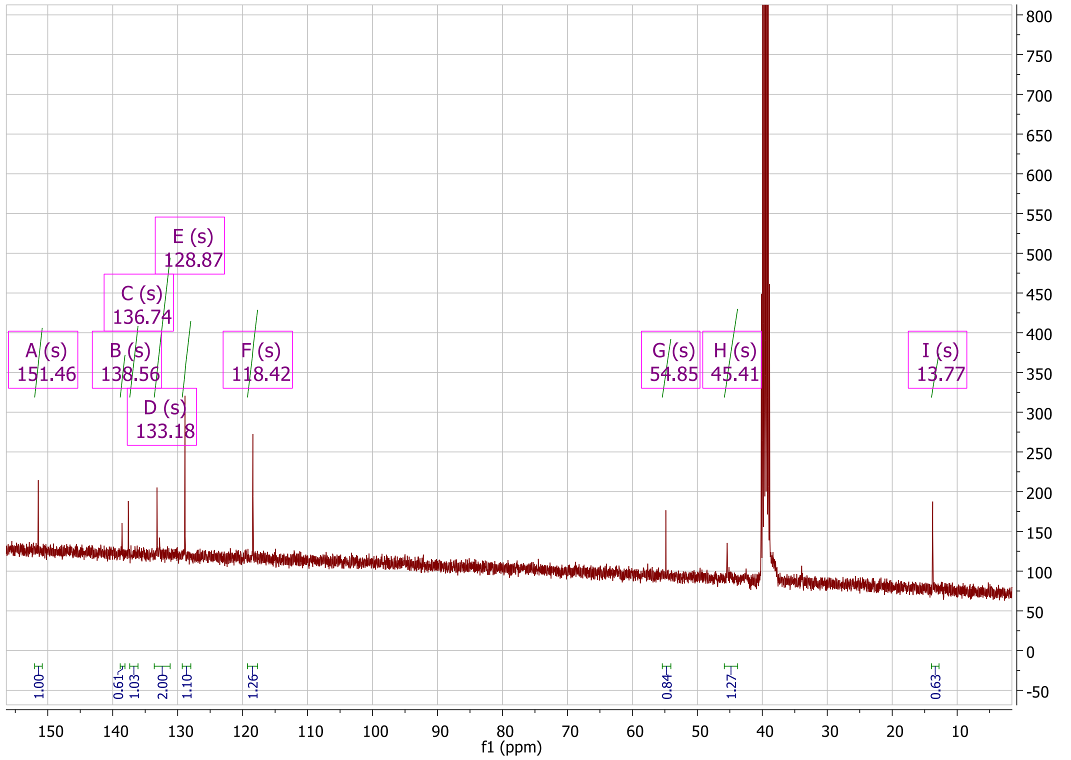

^{1}H NMR spectra of compound **5**

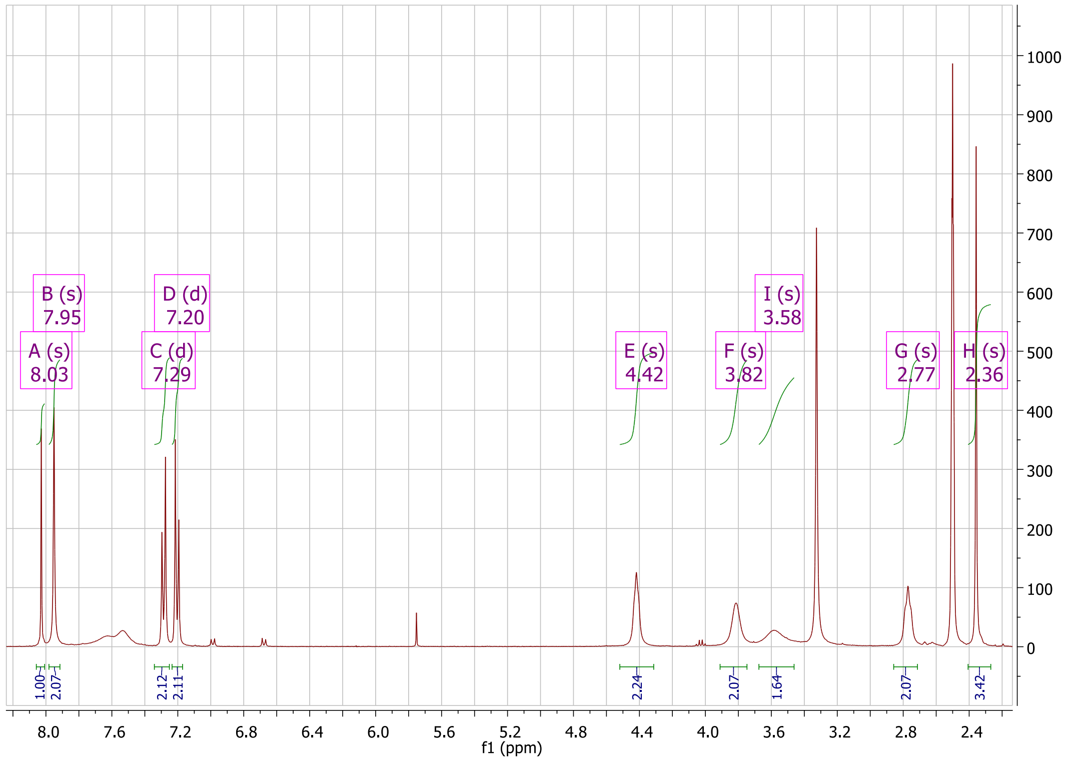

^{13}C NMR spectra of compound **5**

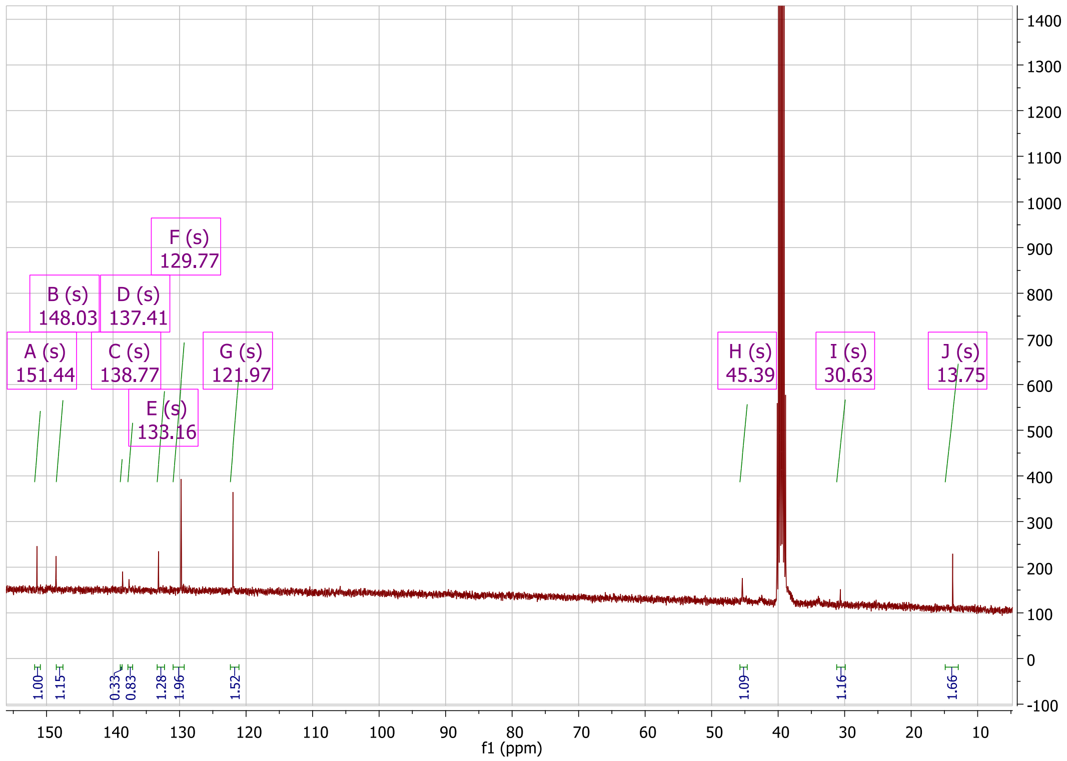

^{1}H NMR spectra of compound **7**

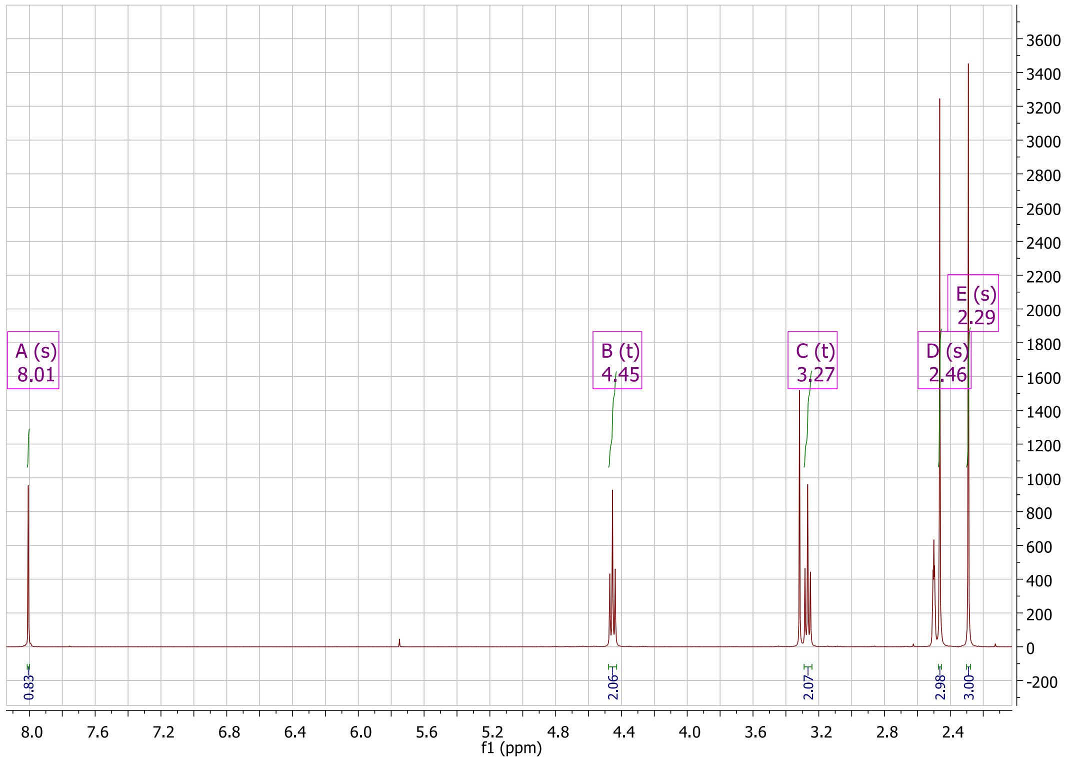

^{13}C NMR spectra of compound **7**

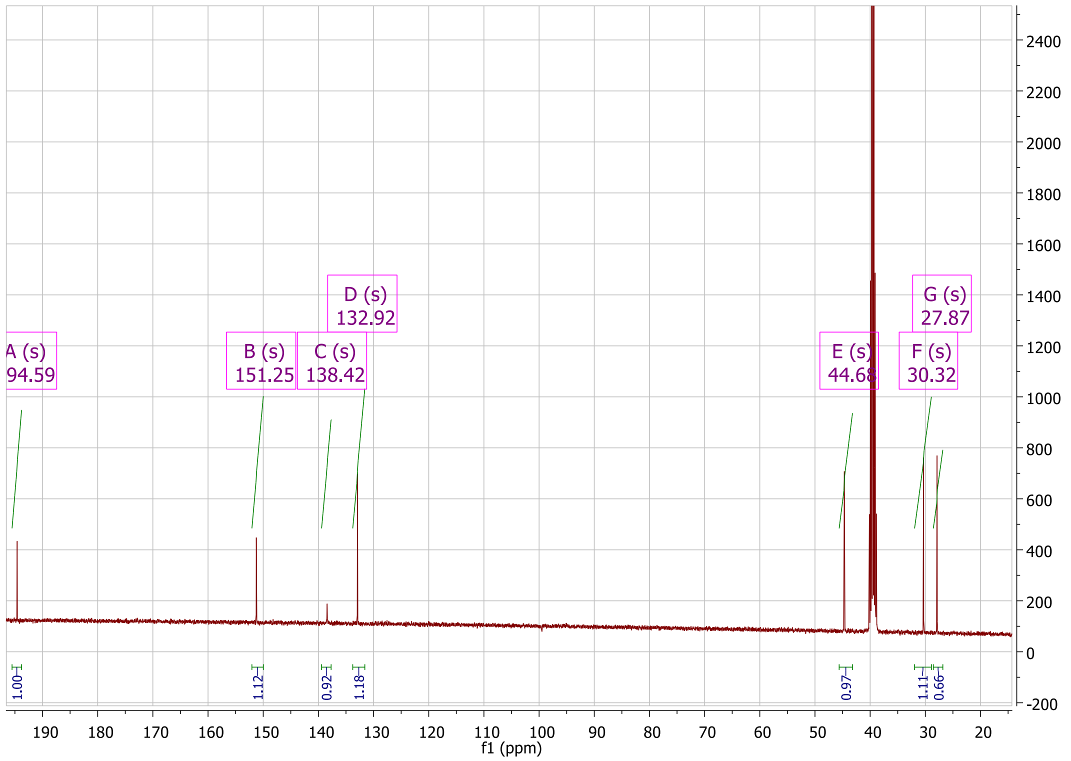

^{1}H NMR of spectra of compound **9**

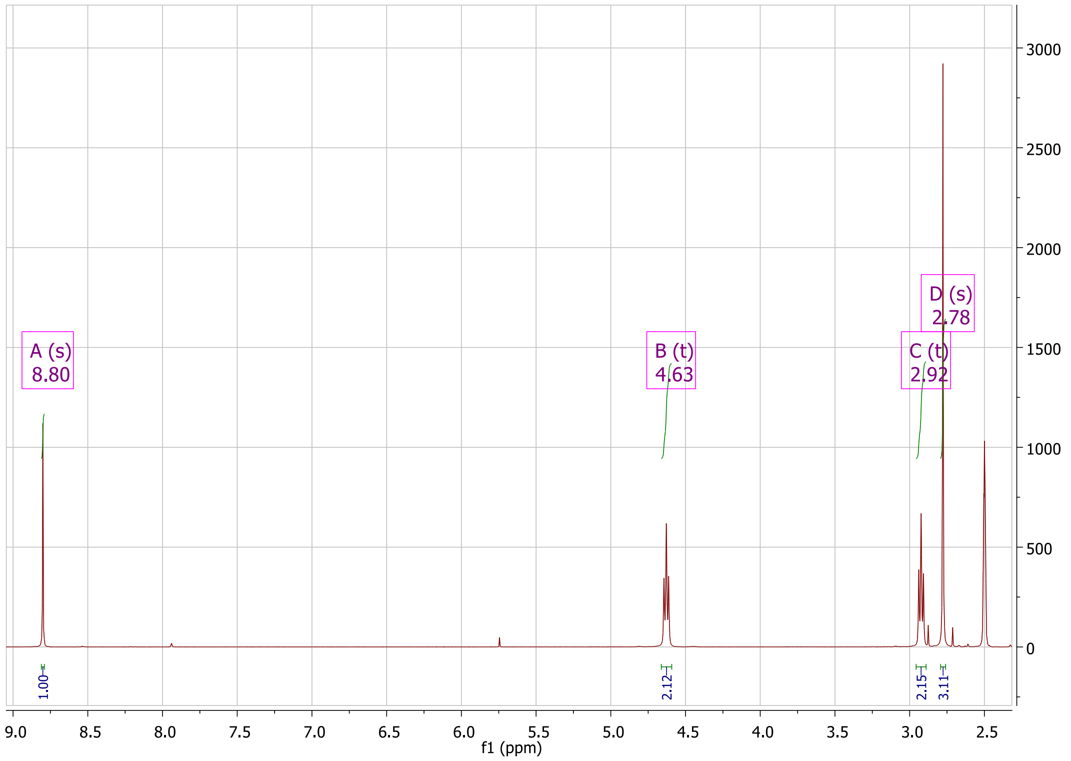

^{13}C NMR spectra of compound **9**

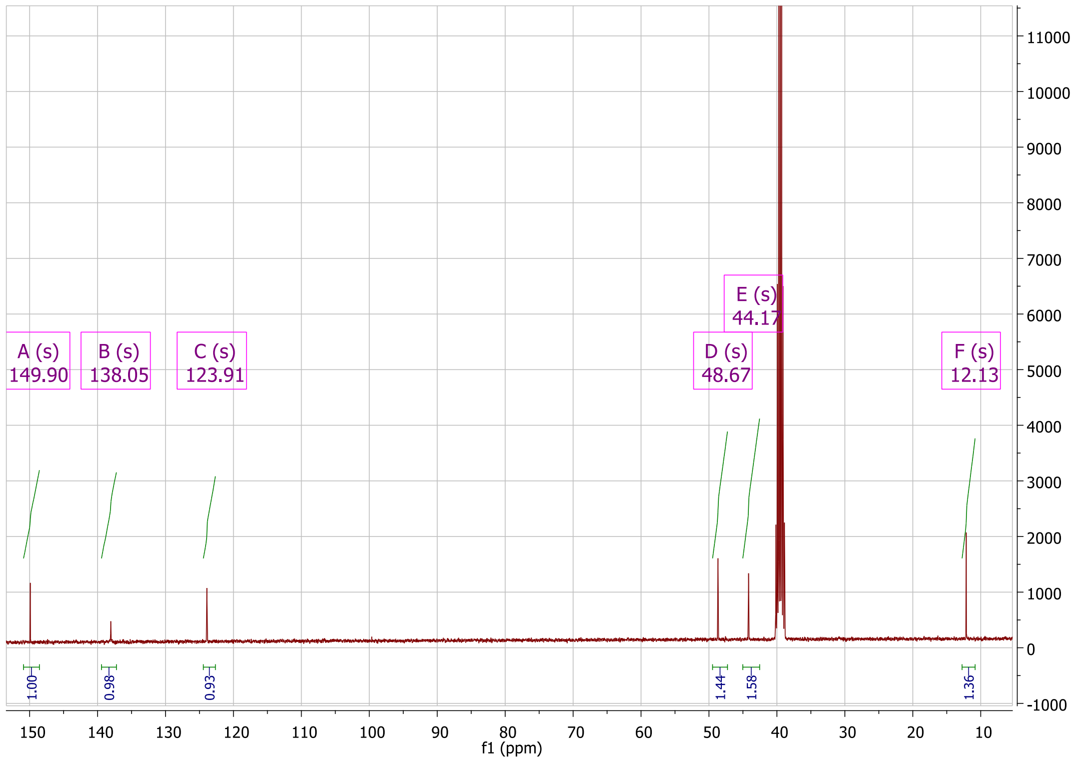

^{1}H NMR spectra of compound **10**

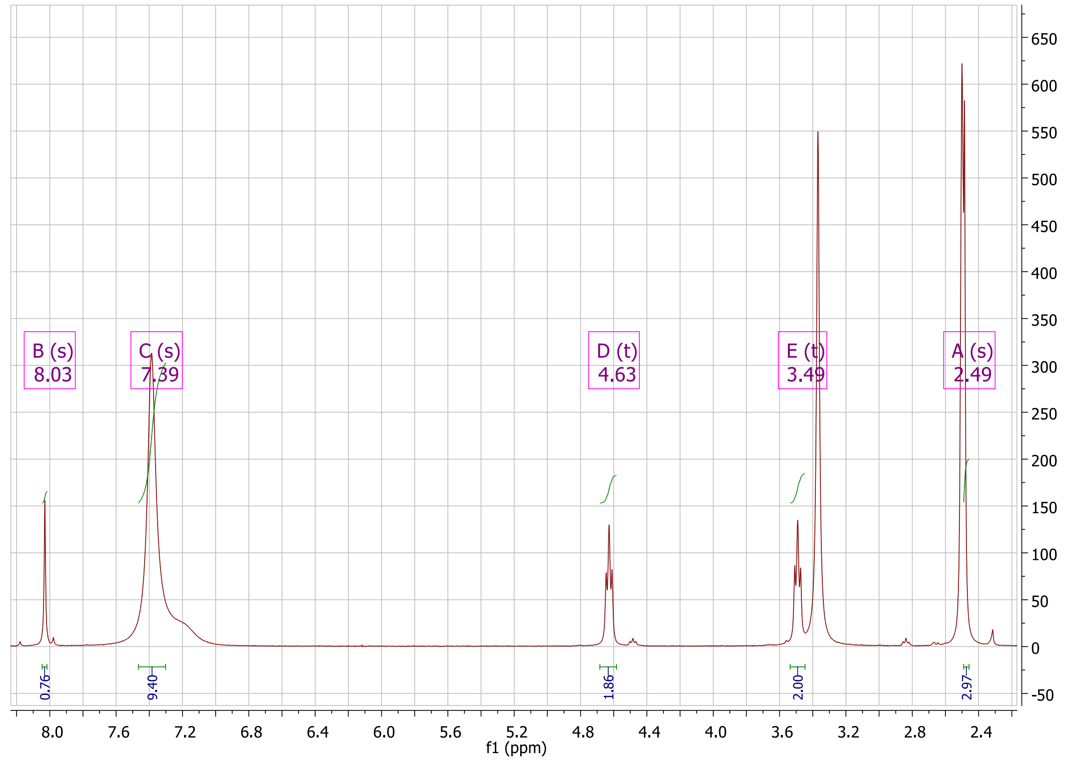

^{13}C NMR spectra of compound **10**

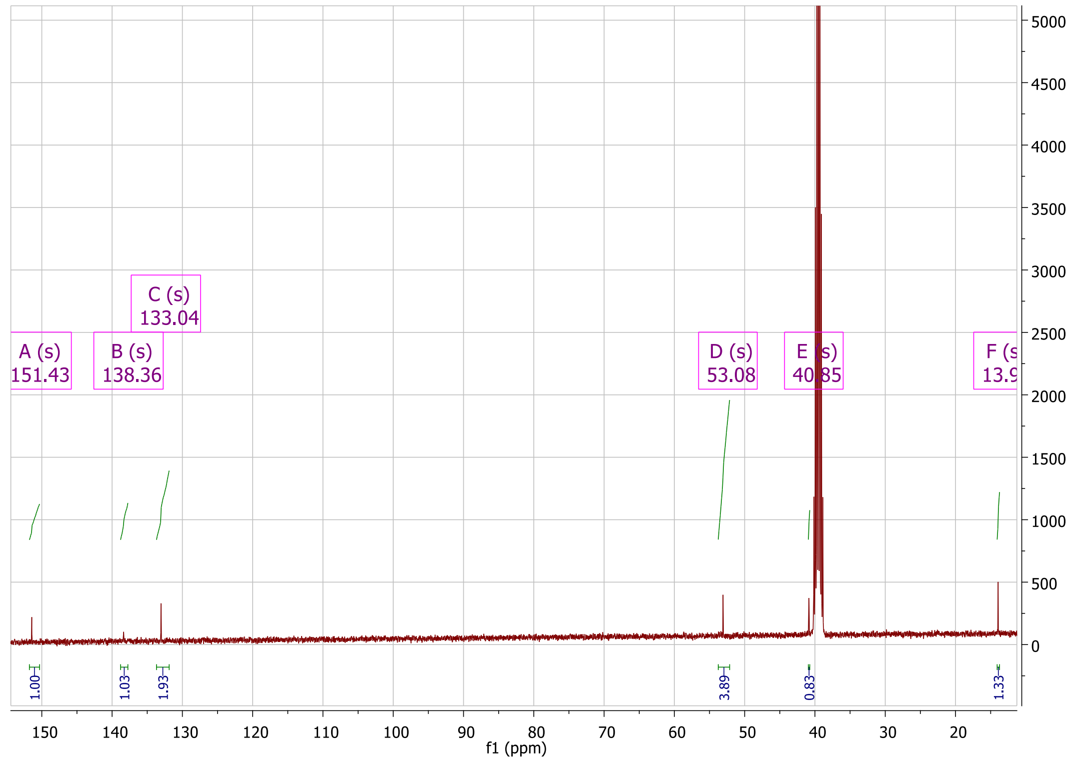

Supplement: Supplemental Material [file IENZ_A_1685510_SM6169.pdf]
